# Triticeal cartilage: the forgotten cartilage

**DOI:** 10.1007/s00276-017-1841-z

**Published:** 2017-03-17

**Authors:** Iain Wilson, J. Stevens, J. Gnananandan, A. Nabeebaccus, A. Sandison, A. Hunter

**Affiliations:** 1 Department of Anatomy, School of Biomedical Sciences, Guy’s Campus, King’s College London, London, SE1 1UL UK; 2 Cardiovascular Division, King’s College London, London, UK; 3 Department of Histopathology, Imperial College Healthcare NHS Trust, London, UK

**Keywords:** Anatomy, Hyoid, Larynx, Thyrohyoid, Triticeal

## Abstract

**Introduction:**

The triticeal cartilage (TC) is a small cartilage found within the thyrohyoid membrane. The TC has a variable prevalence between and within individuals. It has been suggested that absence of a TC results from its failure to separate from the superior horn of the thyroid cartilage (SHTC) and that individuals without a TC will have a longer SHTC. This study aims to identify the prevalence of the TC and investigate the relationship between the length of the SHTC and presence of a TC.

**Methods:**

Eighty seven adult cadavers underwent dissection. Data were collected on presence or absence of a TC and lengths of SHTC.

**Results:**

A TC was identified in 28 cadavers (33%). In cadavers with a unilateral TC, there was no significant difference between the lengths of the SHTC on sides with a TC (1.6 mm, ±SEM 0.12 mm) to sides without a TC (1.7 mm, ±SEM 0.10 mm) (*P* = 0.47). In cadavers with no TCs, the length of the SHTCs (1.8 mm, ±SEM 0.04 mm) was significantly longer than the SHTCs of cadavers with a TC present bilaterally (1.4 mm, ±SEM 0.12 mm) (*P* = 0.02).

**Conclusions:**

A TC was found in 33% of cadavers. This study demonstrates a relationship between the presence of a TC and the length of the SHTC only in cadavers with a TC present or absent bilaterally.

## Introduction

**Fig. 1:**
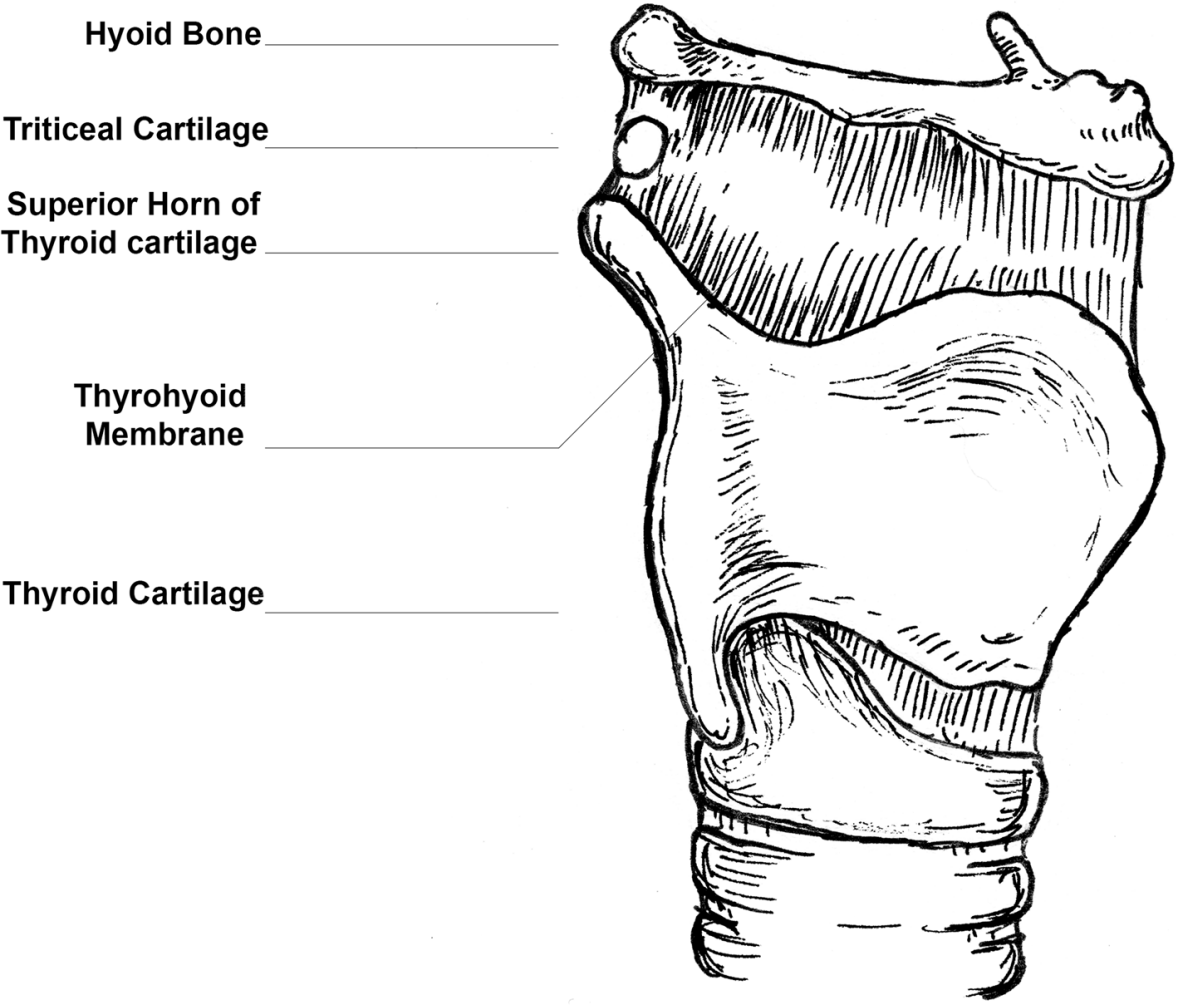
Author’s schematic of the thyrohyoid region showing the triticeal cartilage

The triticeal cartilage (TC) is a small oval-shaped cartilage found within the lateral border of the thyrohyoid membrane between the greater horn of the hyoid bone and the superior horn of the thyroid cartilage (SHTC) (Fig. 1). Like the thyroid and cricoid cartilages, the TC is composed of hyaline cartilage [9]. In a manner similar to the other laryngeal cartilages, the TC demonstrates a tendency to calcify, and in some instances to ossify. The exact pattern of TC calcification is not known, but data from radiographs and cadaveric studies have shown calcification in 5–29% of individuals [1, 7]. The timing of the calcification of the TC is thought to be similar to that of the thyroid cartilage—beginning in the second decade and ending at around 65 years of age [7]. However, this calcification is not directly related to age and considerable variation is observed between individuals. It is unclear if calcification of the TC differs between the sexes [1].

The function of the TC is unknown. Historically, the TC was thought to be the site of attachment for a ‘triticeoglossus muscle (of Bochdalek)’ [3]; however, we have not been able to find any other reports or evidence of this muscle. It has since been suggested that the role of the TC is to strengthen the thyrohyoid ligament [13], although we are not aware of any disadvantage or disability reported by individuals without a TC. An alternate, and in our opinion more likely theory, is that the TC has no function at all in humans [10].

Clinically, there are reports of an enlarged TC causing symptoms such as dysphagia and odynophagia [3] or the sensation of a foreign body [11]. These symptoms are most likely related to compression of an enlarged TC against the laryngopharynx or the nearby internal branch of the superior laryngeal nerve. Awareness of the intimate relationship between the internal branch of the superior laryngeal nerve and the TC is important. It is thought that retraction of this nerve against the TC, in procedures such as carotid endarterectomy and cervical spine operations, could be a focus of nerve injury [14]. There is also one report of an enlarged TC becoming symptomatic after endotracheal intubation [3].

The TC is visible in lateral radiographs of the neck at the level of the third and fourth cervical vertebrae. It is important for clinicians to be able to recognise the cartilage in order to differentiate it from other laryngeal cartilages and pathology of the neck such as foreign bodies and calcified carotid artery atheroma [1].

**Fig. 2:**
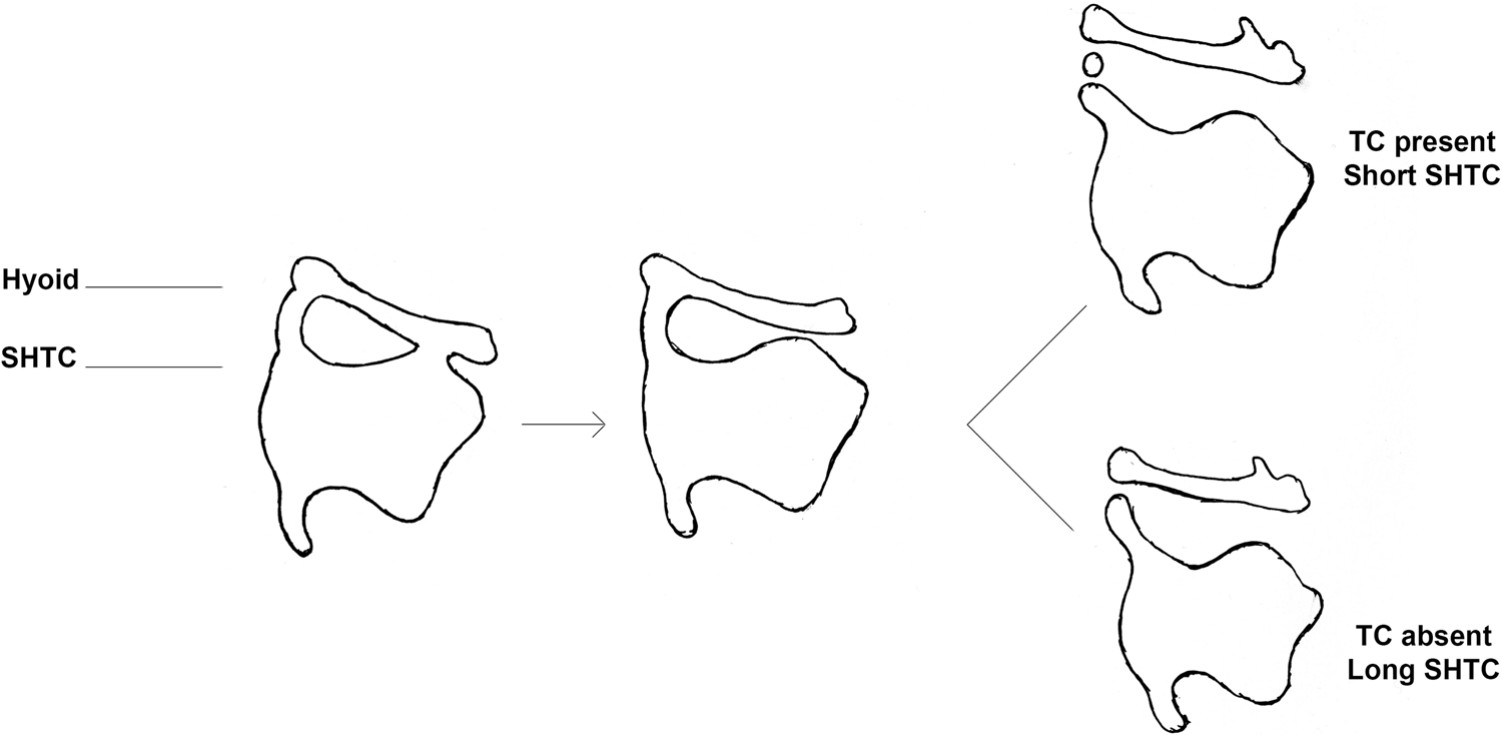
Author’s schematic of the embryological development of the thyrohyoid region as proposed by Grossman [6] (1) The future thyroid cartilage and hyoid are connected the hyothyroid cartilage, (2) the hyoid and larynx separate during development, (3) the hyothyroid separates from the SHTC giving rise to a TC and a short SHTC, (4) or alternately, the hyothyroid cartilage remains attached to the SHTC, so there is absence of a TC but a longer SHTC (4)

The TC is not a constant structure; it may be present unilaterally, bilaterally, or absent. It has been suggested by Grossman [6] that the variable prevalence of the TC can be explained through the embryological development of the neighbouring larynx and hyoid bone. The hyoid and larynx are derived from the pharyngeal arches. The cranial part of the body of the hyoid and lesser horns come from the second arch. The greater horns and caudal part of the body of the hyoid develop from the third pharyngeal arch. The thyroid cartilage is derived from the fourth pharyngeal arch. The future thyroid cartilage and hyoid are connected ventrally at the hypobranchial eminence and dorsally by a cartilaginous bar—the hyothyroid cartilage. At the third month of foetal development, the hyoid and larynx separate. It is proposed that the TC is a remnant of this hyothyroid cartilage, left between the separating hyoid and thyroid cartilage [6]. Grossman [6] also observed from his series of neck radiographs from cadaveric and living subjects that failure of the hyothyroid cartilage to separate from the SHTC leads to the absence of a TC, but a longer residual SHTC (Fig. 2).

The aim of the present work was to establish the prevalence of the TC in a series of cadavers and to investigate the relationship between the presence of the TC and length of the SHTC.

## Methods

Eighty seven adult cadavers from our anatomy department underwent dissection. Cadavers were dissected over a 2-year period. The cadavers had been embalmed in a formaldehyde solution. Dissection was carried out in the anterior triangles of each cadaver to expose the thyrohyoid membrane. The presence or absence of a TC was recorded.

Cadavers dissected in the second year (*n* = 34) underwent measurement of SHTC length (mm), and when present, the length (mm) of the TC was measured. The length of the SHTC was measured from its base (a point on the SHTC level with the superior border of the thyroid cartilage) to its tip (the most superior part of the SHTC). Measurements were made with callipers to the nearest 0.01 mm.

All dissections and measurements were carried out by three investigators. Three independent measurements were taken on each cadaver. The presence or absence of TC and the measurements of the SHTC and TC lengths were only confirmed when all investigators were in agreement.

One cadaver was excluded from the study. This was due to the presence of a prominent unilateral swelling at the tip of an SHTC. We were unable to tell macroscopically if the TC was fused or just adherent to the SHTC. The thyrohyoid region of this cadaver underwent histological assessment. This thyrohyoid region was processed to paraffin wax using routine histological techniques. Sections were cut at 4 μm thick and stained using the usual haematoxylin and eosin and also with Masson’s trichrome used to distinguish cells from surrounding connective tissue more easily. Slides were photographed as whole mount views to better demonstrate the anatomy.

### Data handling

A chi-squared test was used to identify if there was a significant difference between cadaver sex and the presence of a TC. To investigate the relationship between the length of the SHTC and the presence of a TC, the data were divided into two groups. Firstly, data from subjects with a unilateral TC were analysed. The mean lengths of SHTC on the side without a TC acted as a control and were compared with the mean SHTC length on the side with a TC. In the second group, a comparison of SHTC mean length between cadavers with bilaterally absent TC and cadavers with bilaterally present TC was performed. The unpaired student t-test with Welch correction was used to compare differences in SHTC lengths. Finally, where a TC was present, the correlation between its length and the length of the associated SHTC was investigated by means of a Spearman’s correlation coefficient. Statistical significance was accepted at *P* < 0.05.

## Results

The demographics of the two groups of cadavers are set out in Table 1.

**Table 1:** Demographics of the cadavers investigated

	Number of cadavers	Median age (range) in years	Sex M, male; F, female
First year	52	86 (53–103)	32M 20F
Second year	34	82 (60–100)	19M 15F
Totals	86	85 (53–103)	51M 35F

### Prevalence

A TC was found in 33% (28/86) of cadavers. Of the 28 cadavers with a TC, 16 (57%) had a unilateral TC, whereas 12 (43%) had a bilateral TC. Unilateral TCs were found in equal number between right and left sides. Of the 28 cadavers with a TC, 16 were males and 12 were females (Table 2). The *χ* ^2^ test did not identify a significant difference between the presence of a TC and cadaveric sex (*χ* ^2^ value 1.916, DF = 3; *P* value is 0.59).

**Table 2:** Distribution of triticeal cartilages (TCs) according to sex of cadaver

	Number of cadavers	Cadavers with TC	Cadavers without TC	Bilateral	Unilateral
Male	51	16 (31%)	35 (67%)	6 (12%)	10 (20%)
					(5R 5L)
Female	35	12 (34%)	23 (66%)	6 (17%)	6 (17%)
					(3R 3L)
Totals	86	28 (33%)	58 (67%)	12	16 (8R 8L)

### Relationship between presence of TC and SHTC length

There was no significant difference between the mean lengths of the SHTCs whether or not a TC was present (mean difference 0.11 mm, *P* = 0.47). When comparing SHTC mean length between cadavers with no TC present on either side with those that had bilateral TCs, there was a significantly greater mean length in those with no TC present (mean difference 0.33 mm, *P* = 0.02). There was a positive correlation between SHTC length and TC length (*r* 0.41; *P* = 0.05).

### Histological analysis of excluded cadaver

**Fig. 3:**
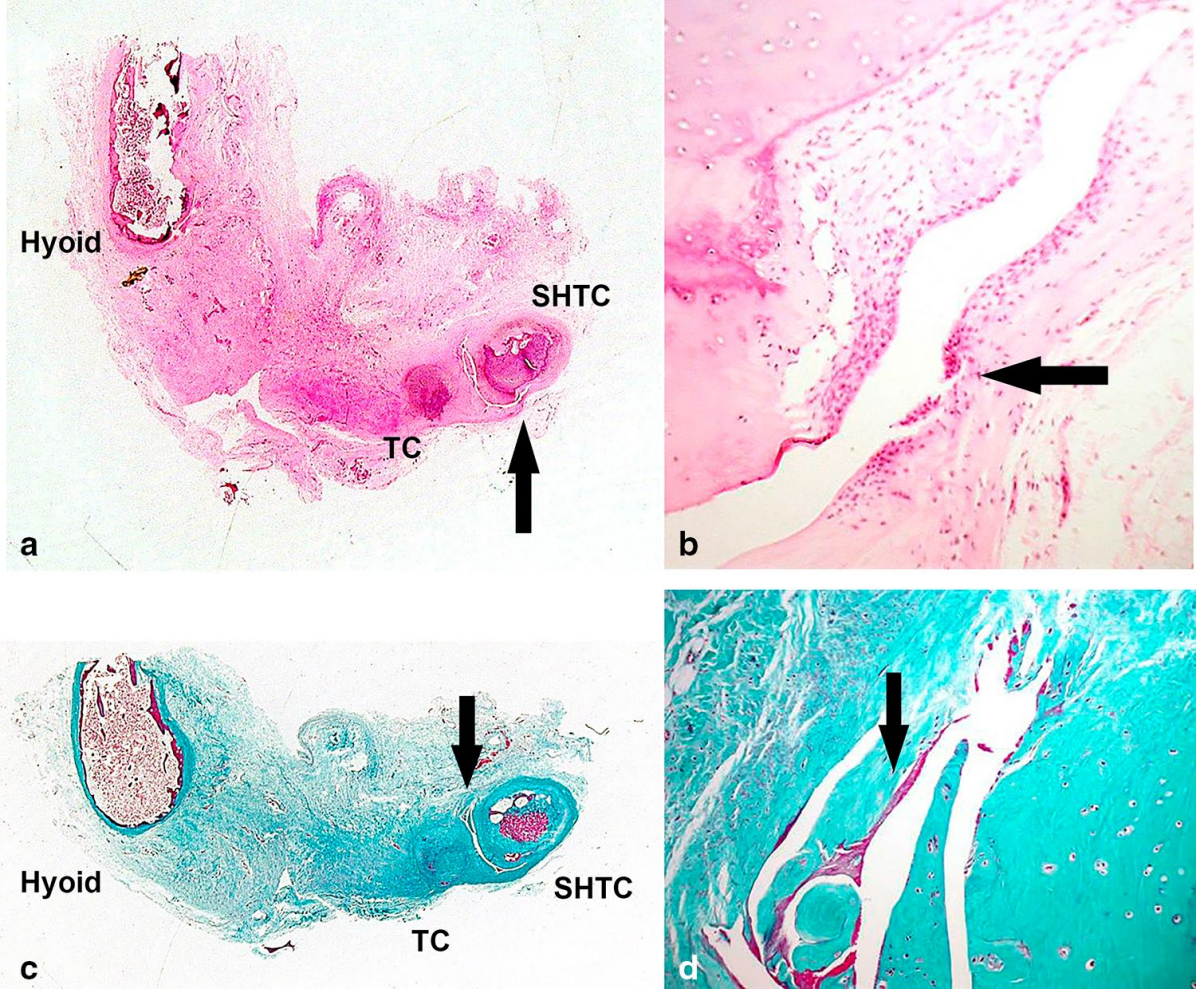
Whole mount views of thyrohyoid apparatus in excluded cadaver. The TC is closely adherent to SHTC, separated by a synovial joint. *Black arrow* indicates synovial lining. Stained using haematoxylin and eosin at ×25 (**a**) and ×100 (**b**) magnification and Masson’s trichrome at ×25 (**c**) and ×100 (**d**) magnification

With regards to the excluded cadaver with an atypically long and bulbous SHTC, microscopic examination of this SHTC revealed this structure to be a distinct TC in close apposition to the SHTC. The TC and SHTC were separated by a synovial membrane (Fig. 3).

## Discussion

### Prevalence

The findings of this study indicate that the presence of the TC varies between individuals. We found the overall prevalence of the TC to be 33%. This finding is supported by two previous studies which observed the TC in 30 and 33% of dissected cadavers [4, 12]. Contrasting results have also been reported—one study in 40 dissected Nigerian cadavers observed a prevalence of 13.5% [2], whereas a much higher prevalence of 65% was seen from a study of dissections in 232 Japanese cadavers [15]. A study to investigate ethnic variation in TC prevalence would be an interesting area for further research.

### Laterality of the TC

We have shown that when a TC is found, it is more likely to be unilateral than bilateral (16 unilateral, 12 bilateral). One previous study found the opposite, demonstrating a tendency for bilateral cartilages (bilateral 17.5%, unilateral 12.5%) [15]. Furthermore, one study reports that TCs in all cases were found to be bilateral [12].

### Sex

There was no significant difference in the prevalence of the TC between male and female cadavers. This supports the findings of other studies which report no clear pattern of sex predilection [2, 4, 12], with the exception of one study which reports the TC to be found four times more frequently in male versus female Japanese cadavers [15]. The authors of the latter study were unable to explain why a sex difference was present.

### Length of SHTC

It has been proposed that the TC is derived from a hyothyroid cartilage which joins the SHTC and the hyoid bone. During development, the hyothyroid cartilage may remain attached to or separate from the SHTC. It is hypothesised that if the hyothyroid cartilage remains attached to the SHTC, there will be no TC present, and the SHTC tends to be longer. Alternatively, if the hyothyroid cartilage separates from the SHTC, a TC will be present with a relatively short SHTC [6]. In the current study, this pattern was observed, but only in cadavers with bilateral TCs and not in those with unilateral TCs. In addition, we observed that TC length tended to be positively correlated to SHTC length. The opposite relationship between TC and SHTC length would be expected, were we to accept Grossman’s theory.

We are unsure as to why a relationship between presence of TC and SHTC length was seen only in cadavers with bilaterally present TCs. One reason could be due to the inherent limitations of our study. We did not record the height of each cadaver or length of the laryngeal skeleton. In particular, the length of the greater horn of the hyoid bone was not recorded. These measurements may have confounded the relationship between presence of TC and length of SHTC. It is also possible that we may have missed small or even microscopic TCs. Finally, these measurements were only recorded on 34 cadavers, and a larger study would have more power to support or reject the relationship between presence of a TC and SHTC length.

Grossman’s original study [6] must also be interpreted in light of its own limitations. Firstly, only subjects with ossified TCs visible on radiographs were included. Individuals with unossified TCs or TCs not visible on radiographs would therefore not have been included in the analysis. Secondly, it is not stated how many subjects were included in the study. Further to this, a large study using data obtained from dissection was unable to demonstrate a relationship between the presence or absence of a TC and length of SHTC [15]. Considering the findings of the latter study, the disparate results of the present study and the flaws in Grossmans study [6]; we cannot fully accept that there is a relationship between the presence of a TC and length of the SHTC.

Although this study cannot explain the variable prevalence of the TC, the idea of a persistent hyothyroid cartilage forming a TC remains an attractive theory. The idea for this theory comes from observing anatomical variations in humans and a structure called the hyoid apparatus seen in some adult mammals.

Two cases of cartilaginous and bony structures joining the SHTC and the greater horn of the hyoid bone have been reported in adult humans [3, 8]. These structures share a remarkable similarity to the hyothyroid cartilage described by Grossman [6]. We feel it is possible to suggest that these structures could represent a ‘persistent hyothyroid cartilage’ that has failed to regress into a TC.

**Fig. 4:**
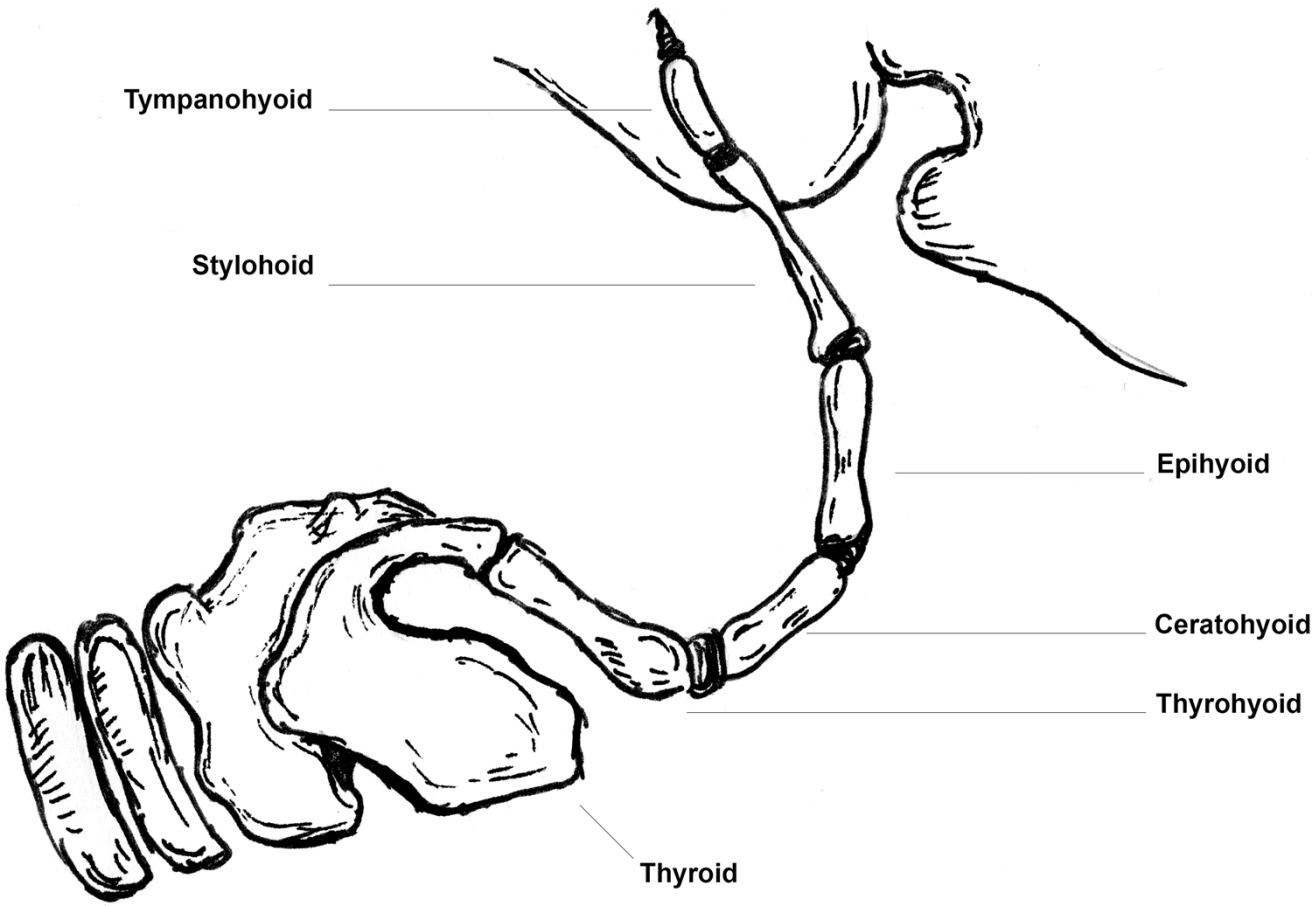
Author’s schematic of one side of a dog’s hyoid apparatus. The component parts from proximal to distal are the tympanohyoid, stylohyoid, epihyoid, ceratohyoid, thyrohyoid. The basihyoid which links each side is not pictured

Taking this theory further, we feel the hyothyroid cartilage and the structures in the above two cases share a crude similarity with a structure called the hyoid apparatus seen in some adult mammals. The hyoid apparatus is a bony structure composed of five paired bones and one unpaired bone linking the skull to the SHTC. The bony components of the hyoid apparatus are united by cartilaginous joints [5] (Fig. 4). Although entirely speculative, it could well be that the hyothyroid cartilage and TC represent an atavistic remnant of the hyoid apparatus.

We feel that the similarity of the hypothyroid cartilage to the hyoid apparatus goes even further. In the hyoid apparatus the articulation between the SHTC and the most caudal part of the hyoid apparatus, the thyrohyoid, can be through either cartilaginous or synovial joints. Interestingly, in the two cases that report a structure joining the hyoid and SHTC in humans—the articulations to the SHTC are synovial and cartilaginous respectively [3, 8] Further to this, in the present study the cadaver sent for histological assessment was found to have a TC articulating with the SHTC via a synovial joint (Fig. 3). Further study of the hyoid apparatus in other animals could provide more clues as to the origins of the TC.

## Conclusions

This study reports the prevalence of the TC to be 33%. Given the absence of a uniform relationship between the TC and SHTC, we cannot provide support for the idea that the TC arises from the SHTC, leaving behind a shorter SHTC.

Why the TC demonstrates variability between and within individuals is unclear. With no known selection advantage of having a TC, the variability of the TC could be due to chance. Research from larger studies, developmental studies, and comparative anatomy may be able to provide better understanding of this forgotten cartilage of the larynx.
